# Cross-species transcriptomics reveals differential regulation of essential photosynthesis genes in *Hirschfeldia incana*

**DOI:** 10.1093/g3journal/jkae175

**Published:** 2024-08-08

**Authors:** Francesco Garassino, Sofia Bengoa Luoni, Tommaso Cumerlato, Francisca Reyes Marquez, Jeremy Harbinson, Mark G M Aarts, Harm Nijveen, Sandra Smit

**Affiliations:** Laboratory of Genetics, Wageningen University & Research, Droevendaalsesteeg 1, Wageningen 6708 PB, The Netherlands; Laboratory of Genetics, Wageningen University & Research, Droevendaalsesteeg 1, Wageningen 6708 PB, The Netherlands; Laboratory of Genetics, Wageningen University & Research, Droevendaalsesteeg 1, Wageningen 6708 PB, The Netherlands; Laboratory of Genetics, Wageningen University & Research, Droevendaalsesteeg 1, Wageningen 6708 PB, The Netherlands; Laboratory of Biophysics, Wageningen University & Research, Stippeneng 4, Wageningen 6708 WE, The Netherlands; Laboratory of Genetics, Wageningen University & Research, Droevendaalsesteeg 1, Wageningen 6708 PB, The Netherlands; Bioinformatics Group, Wageningen University & Research, Droevendaalsesteeg 1, Wageningen 6708 PB, The Netherlands; Bioinformatics Group, Wageningen University & Research, Droevendaalsesteeg 1, Wageningen 6708 PB, The Netherlands

**Keywords:** photosynthesis, transcriptomics, light-use efficiency, Brassicaceae, panproteome, Plant Genetics and Genomics

## Abstract

Photosynthesis is the only yield-related trait not yet substantially improved by plant breeding. Previously, we have established *H. incana* as the model plant for high photosynthetic light-use efficiency (LUE). Now we aim to unravel the genetic basis of this trait in *H. incana*, potentially contributing to the improvement of photosynthetic LUE in other species. Here, we compare its transcriptomic response to high light with that of *Arabidopsis thaliana*, *Brassica rapa*, and *Brassica nigra*, 3 fellow Brassicaceae members with lower photosynthetic LUE. We built a high-light, high-uniformity growing environment, in which the plants developed normally without signs of stress. We compared gene expression in contrasting light conditions across species, utilizing a panproteome to identify orthologous proteins. In-depth analysis of 3 key photosynthetic pathways showed a general trend of lower gene expression under high-light conditions for all 4 species. However, several photosynthesis-related genes in *H. incana* break this trend. We observed cases of constitutive higher expression (like antenna protein *LHCB8*), treatment-dependent differential expression (as for *PSBE*), and cumulative higher expression through simultaneous expression of multiple gene copies (like *LHCA6*). Thus, *H. incana* shows differential regulation of essential photosynthesis genes, with the light-harvesting complex as the first point of deviation. The effect of these expression differences on protein abundance and turnover, and ultimately the high photosynthetic LUE phenotype is relevant for further investigation. Furthermore, this transcriptomic resource of plants fully grown under, rather than briefly exposed to, a very high irradiance, will support the development of highly efficient photosynthesis in crops.

## Introduction

Considering the projected global population growth, the increasing effects of global warming, and the need for a more sustainable means of production, it is evident that the agricultural sector is under substantial pressure to increase crop yields while reducing land use and inputs such as fertilizers and pesticides. Over the past decade photosynthesis has taken a central role in plant research aimed at increasing crop yields because it plays a major role in the crop energy conversion efficiency, the only yield-related trait of food and feed crops that has not yet been maximized or even substantially improved by plant breeding (Zhu *et al*. 2010).

While increasing crop productivity via improved photosynthetic efficiency was proposed over 40 years ago (Austin 1989), limited results have been achieved so far due to the physiological and genetic complexity of the photosynthetic process. Studies based on modeling of the photosynthetic process, bottleneck identification, and genetic modification aimed at overcoming identified bottlenecks have proven successful in some field crops, with yield increases ranging between 15% and 28% (Kromdijk *et al*. 2016; López-Calcagno *et al*. 2019, 2020; Yoon *et al*. 2020; De Souza *et al*. 2022). However, the inconsistency of results over multiple seasons (De Souza *et al*. 2022) and across species (Garcia-Molina and Leister 2020) or growing conditions (Ruiz-Vera *et al*. 2022) indicates that more knowledge is needed on photosynthetic processes and how they are influenced by the environment across a range of timescales if we are to systematically increase the photosynthetic efficiency in crops.

Plant photosynthesis is defined as the process in which energy from light radiation is converted into chemical energy via a complex series of reactions resulting in the production of carbohydrates and oxygen (Salisbury and Ross 1992). A first set of photosynthetic reactions, catalyzed by photosystem complexes and an associated electron transport chain, is responsible for converting light radiation energy into chemical energy. This chemical energy is then stored in metabolically useful reducing agents (e.g. NADPH) and the energy-rich phosphate donor ATP. The processes linking light absorption to the formation of ATP and NADPH and other reducing agents collectively form the light reactions of photosynthesis (Schulze and Caldwell 1995). The energy-rich reducing agents and ATP then drive a second set of photosynthetic reactions, the so-called dark reactions. These, generally referred as to the Calvin–Benson cycle, result in the conversion of the inorganic carbon substrate $CO_{2}$ into organic carbohydrate molecules (Schulze and Caldwell 1995). Bottlenecks or constraints—sites whose modification could result in improved photosynthesis—have been identified in both the light and dark reactions (Zhu *et al*. 2010). Furthermore, bottlenecks affecting photosynthesis have been identified in processes that would not be defined as strictly photosynthetic, such as the diffusion of $CO_{2}$ into and through leaves to the site of $CO_{2}$ fixation in chloroplasts, and the transport of carbohydrates from photosynthetically active cells to carbon sinks elsewhere in the plant (Singh *et al*. 2014).

Our current knowledge of the key mechanisms and components of photosynthesis is the result of decades of studies in plants and other photosynthetic organisms (Johnson 2016). This amounts to a vast body of knowledge, but on its own it is insufficient to improve crops’ photosynthesis and their yield. Studies conducted so far have highlighted how the link between crop photosynthesis and productivity is much more complex than originally thought, as a result of interactions of this process with plant development and environmental factors (Araus *et al*. 2021). One characteristic of photosynthesis that can have a major impact on crop productivity is the decreasing light-use efficiency (LUE) that occurs with increasing irradiance, giving rise to the light-saturation of photosynthesis and limitation of assimilation rate. This limitation has a substantial impact on productivity at irradiance levels normally recorded during summer in temperate areas of our planet. We define photosynthetic LUE as the ratio between photosynthetically assimilated $CO_{2}$ and incident light radiation, or irradiance. The decrease in LUE due to increasing irradiance is well-known and its causes are linked to both limitations in the photosynthetic process and other associated processes (Taylor *et al*. 2023).

Evidence has been reported for large natural variation in photosynthesis rates, and therefore photosynthetic LUE, among crop and other plant species (Theeuwen *et al*. 2022; Yin *et al*. 2022). This suggests that a degree of plasticity exists for photosynthesis that could be leveraged to increase the photosynthesis of crop species. However, it is nowadays clear that increases might only be achieved if knowledge is accumulated on the regulation of the photosynthetic process as well as specific strategies some plant species might have evolved that result in photosynthesis optimized to meet unusual goals (Taylor *et al*. 2023). One powerful way to map the genetic basis of complex biological processes is via the analysis of the associated transcriptional activity. Over the past years, several studies on transcriptional activity in a number of species have increased our knowledge on the response of photosynthesis to irradiances of different intensities or changes in irradiance. It was shown that *Arabidopsis thaliana* acclimates to high light by increasing expression of heat-shock response genes and lipid-remodeling genes (van Rooijen *et al*. 2018), that rice (*Oryza sativa*) exposed to variations in irradiance associated to field conditions activates a large number of biotic and abiotic stress genes (Hashida *et al*. 2022), and that barley (*Hordeum vulgare*) expresses genes involved in phenolic compounds accumulation at a higher level with increasing irradiance (Pech *et al*. 2022). However, none of these studies applied a long-term, very high irradiance treatment, similar to what is experienced by plants growing in natural temperate environments during summer months, at high altitude conditions, or in the equatorial region. Neither did they include species with a particularly high photosynthetic LUE. We consider these 2 factors essential for unraveling the physiological and genetic basis of photosynthetic LUE, and for ultimately building more light-use efficient photosynthesis in our crops (Taylor *et al*. 2023).

Here, we present the analysis of gene expression in *Hirschfeldia incana* (L.) Lagr.-Foss., the species we previously proposed as preferred model for studies on high photosynthetic LUE due to its unique combination of photosynthetic performance, accessible genetics, membership to the well-studied Brassicaceae plant family, and easy growing and reproduction in laboratory conditions (Garassino *et al*. 2022; Taylor *et al*. 2023). The gene expression under contrasting high light (HL) and low light (LL) irradiance conditions is compared to that of Brassicaceae family relatives *A. thaliana*, *Brassica rapa*, and *Brassica nigra*. While *A. thaliana* does not share the whole-genome triplication that the other 3 species underwent and is, therefore, more distantly related, *B. rapa* and *B. nigra* represent the different evolutionary history of 2 major lineages emerging after this event (Arias and Pires 2012). Using transcriptomics we aim to elucidate which genes and thus pathways are involved in the maintenance of a high photosynthetic LUE at high irradiance in *H. incana*. First, we describe the experiment we conducted under high irradiance, and present the results of differential gene expression (DGE) analysis performed on each of the 4 species. Then, we report on the use of a panproteome to compare gene expression changes across the 4 species, and on the exploration of common and divergent trends in the gene expression response to high-light by means of untargeted enrichment analyses. We then present the results of targeted analysis of expression patterns across the 4 species for genes involved in key photosynthesis-related pathways. Lastly, we discuss the findings in the light of their implications for *H. incana’s* higher photosynthetic LUE at high irradiance. Our work thus describes the transcriptional differences associated with plant growth under highly contrasting irradiance conditions, and serves as a resource for the elucidation of the genetic determinants of the striking photosynthetic capacity of *H. incana*.

## Materials and methods

### Construction of high-uniformity growth system

Two custom light ceilings were built for this study. Each ceiling measured $∼4.3m^{2}(l\;175\;\mathrm{cm},w\;245\;\mathrm{cm})$, was equipped with 6 dimmable VYPR2p LED fixtures (Fluence, Austin, USA) arranged in 3 equally spaced rows (between-rows distance of 60cm, and was hung so that fixtures would be 1 m high over plants. We then centered 2 custom-made growing tables measuring $∼1.6m^{2}(l\;118\;\mathrm{cm},w\;137\;\mathrm{cm})$ under the custom light ceilings, divided each table into 30 growing areas, each measuring $∼0.05\;m^{2}$, and performed irradiance measurements at the center of each growing area. By calculating averages over the 30 areas under each light ceiling, we optimized the output of the LED fixtures to have average irradiances as close as the reference values we chose for our treatments.

### Plant material and growing conditions

*Hirschfeldia incana* accession “Nijmegen”, *B. nigra* accession “DG1”, *B. rapa* R-o-18, and *A. thaliana* Col-0 were used for this experiment. “Nijmegen” is an inbred line (over 6 rounds of inbreeding) from an *H. incana* plant originally collected in Nijmegen, The Netherlands; “DG1” is a second-generation inbreeding line of *B. nigra* sampled from a natural population near Wageningen, The Netherlands; and “R-o-18” is a *B. rapa* inbred line (Stephenson *et al*. 2010; Bagheri *et al*. 2012).

Seeds of all species were germinated on a peat-based potting mix for 9 days under an irradiance of $200\;\mu \mathrm{mol}\;m^{-2}\;s^{-1}$. Twelve healthy seedlings per species were then transferred to 2L pots(∅13.9cm,h17.4cm, Soparco, Condé-sur-Huisne, France) filled with a peat-based potting mixture enriched with perlite and 2.5g/L Osmocote® Exact Standard 5–6M slow-release fertilizer (ICL Specialty Fertilizers, Geldermalsen, The Netherlands).

Plants were germinated and grown in a climate-controlled room equipped with the custom arrays of high-output LED light modules described above, with a photoperiod of 12 h day and 12 h night, and air temperature set at 23 °C and 20 °C, respectively. Humidity and $CO_{2}$ levels were set at 70% and 400 ppm. Six plants per species were assigned to the HL treatment of $1,800\;\mu \mathrm{mol}\;{\text{m}}^{-2}\;{\text{s}}^{-1}$ (measured irradiance average: $1843.6\;\mu \mathrm{mol}\;{\text{m}}^{-2}\;{\text{s}}^{-1}$ ) and the remaining 6 to the LL treatment of$\;200\;\mu \mathrm{mol}\;{\text{m}}^{-2}\;{\text{s}}^{-1}$ (measured irradiance average:$\;227.5\;\mu \mathrm{mol}\;{\text{m}}^{-2}\;{\text{s}}^{-1}$). Irradiance uniformity was very high for both HL and LL treatments, with a ${\text{U}}_{2}$ value (defined as *minimum irradiance/maximum irradiance*, Sun *et al*. 2014; Hu *et al*. 2015) of 0.93. Plant positions were randomized across growing areas. Plants assigned to the LL treatment were fertigated daily, while plants assigned to the HL treatment were fertigated twice a day, with a custom nutrient solution $(0.6\;\mathrm{mM}\;NH_{4}^{+},3.6\;\mathrm{mM}\;K^{+},2\;\mathrm{mM}\;Ca^{2+},$  $0.91\;\mathrm{mM}\;Mg^{2+},6.2\;\mathrm{mM}\;NO_{3}^{-},1.66\;\mathrm{mM}\;SO_{4}^{2-},0.5\;\mathrm{mM}\;P,35\;\mu M\;Fe^{3+},$  $8\;\mu M\;Mn^{2+},5\;\mu M\;Zn^{2+},$  $20\;\mu M\;B,0.5\;\mu M\;Cu^{2+},0.5\;\mu M\;Mo^{4+})$.

### Sampling and RNA extraction

Twenty-eight days after sowing, samples representative of the whole canopy were collected from all plants. All leaves (for smaller plants such as *A. thaliana* and *H. incana*, especially when grown under LL) or half the total number of leaves were excised from plants, transferred to 50mL tubes, and flash-frozen in liquid nitrogen. All leaf samples were subsequently crushed with a mortar and pestle in excess liquid nitrogen, and further homogenized with glass beads for 2min at 30Hz in a MM300 Mixer Mill (Retsch GmbH, Haan, Germany). Total RNA was extracted with the RNeasy Plant Mini Kit (QIAGEN N.V., Venlo, The Netherlands) according to manufacturer’s instructions, and eluted using 50μL of DNAse/RNAse-free water. The following DNAse treatment and RNA recovery were performed as described in Oñate-Sánchez and Vicente-Carbajosa (2008). Six microliters of 10× DNAse buffer and 4μL of RQ1 DNAse (Promega, Leiden, The Netherlands) were added to 50μL of RNA, and incubated for 30 min at 37 °C. The RNA was then precipitated overnight using ammonium acetate and ethanol, and resuspended in 25μL of DNAse/RNAse-free water. To check RNA quality and integrity, 1μL of RNA was used to (1) load a 1% agarose-Ethidium bromide gel and after electrophoresis observe the bands using standard imaging and (2) to determine spectrophotometric parameters with a Nanodrop 2000 (Thermo Fisher Scientific Inc., Waltham, USA). The RNA was further quantified using the Qubit RNA BR Assay kit and a Qubit 4 fluorometer (Thermo Fisher Scientific Inc.).

### Sequencing

RNA from 5 of the 6 plants of each species grown under each light treatment was sequenced by Novogene (UK) Company Ltd., Cambridge, UK. Poly-A enriched RNA was employed to prepare sequencing libraries with the NEBNext® Ultra^TM^ RNA Library Prep Kit (New England Biolabs, Ipswich, USA). Paired-end, 150-bp-long reads (PE150) were generated with a NovaSeq 6000 system (Illumina Inc., San Diego, USA) aiming at obtaining 6 Gb of data per sample.

### Selection and preparation of genome assemblies and annotations

For mapping of sequencing reads and quantification of gene expression, the TAIR10 (Swarbreck *et al*. 2008) genome assembly and the Araport11 annotation (Cheng *et al*. 2017) were used for *A. thaliana*, the “Chiifu” v3.0 assembly and annotation (Zhang *et al*. 2018) were used for *B. rapa*, the “Ni100” v2.0 assembly and annotation (Perumal *et al*. 2020) were used for *B. nigra*, and the “NIJ6” v1.0 assembly and annotation (Garassino *et al*. 2022) were used for *H. incana*. For panproteome building, the v3.0/3.1 *A. arabicum* (Fernandez-Pozo *et al*. 2021), the v1.0 *Raphanus raphanistrum* (Moghe *et al*. 2014), the v1.0 *Raphanus sativus* (Kitashiba *et al*. 2014), and the v1.0 *Sisymbrium irio* (Haudry *et al*. 2013) genome assemblies and annotations were employed together with the aforementioned ones. The exact locations where the various files were downloaded from can be found in Supplementary Table S13.

Statistics were collected for all genome assemblies and annotations with custom Python (v3.11.0) scripts and are reported in Supplementary Table S14. Given that not all genome annotations contained multiple transcript isoforms, all General Feature Format (GFF) files were processed with the agat_sp_keep_longest_isoform.pl script from the AGAT toolkit v1.0.0 (Dainat 2021) to generate annotations containing only the longest transcript isoforms of all gene models. Subsequently, these GFF files were filtered with the agat_sp_filter_by_ORF_size.pl script to remove all gene models that would have yielded protein sequences shorter than 30 amino acids. Finally, a number of gene models identified in the *R. raphanistrum* and *R. sativus* annotation that would still not result in protein sequences (due to stop codons embedded in their sequence) were removed from the corresponding annotations with the agat_sp_filter_feature_ from_kill_list.pl script. The resulting filtered annotation files are provided with the data package linked to this article.

### Identification and analysis of differentially expressed genes

The quality of sequencing libraries was assessed with MultiQC (Ewels *et al*. 2016) v1.11. A snakemake (v7.19.1) (Mölder *et al*. 2021) pipeline was employed to automate subsequent read mapping and transcript quantification steps. Reads were aligned to reference genome assemblies with 2 passes of the STAR (Dobin *et al*. 2013) v2.7.10a aligner (STAR indexing running with parameters –sjdbOverhang 139 and –genomeSAindexNbases 13, STAR aligner running with parameter –clip5pNbases 10 10). Assembly and quantification of full-length transcripts were then achieved with StringTie (Pertea *et al*. 2015) v2.2.1 (running with option –e). Per-sample gene and transcripts counts were then grouped by species with the prepDE Python script included in the StringTie suite (running with parameter –l 140). Transcripts per million (TPM) counts (Wagner *et al*. 2012) were extracted for visualization purposes from the StringTie output with a custom Python script.

Relationships between samples of the same species were explored with PCA plots of transcript counts transformed by means of regularized logarithm (Love *et al*. 2014). Differentially expressed genes (DEGs) were subsequently identified with DESeq2 (Love *et al*. 2014) v1.34.0 running in R (R Core Team 2021) v4.1.1.

### Panproteome construction

Proteomes were created from the filtered annotations of all 8 species with the AGAT toolkit agat_sp_extract_sequences.pl script, running with options –p, –cis, and –cfs. A panproteome was subsequently constructed by running PanTools v4.1.0 (Sheikhizadeh Anari *et al*. 2018; Jonkheer *et al*. 2022) commands build_panproteome, busco_protein, (with options –if brassicales_odb10 –version busco4), optimal_grouping, and change_grouping (with option –version 4, and thus running with a relaxation parameter of 4). A separate panproteome was constructed featuring chloroplast proteomes for *A. thaliana*, *B. rapa*, *B. nigra*, and *H. incana* with PanTools commands build_panproteome and group (with the same relaxation parameter of 4). The panproteome was visualized by making UpSet plots (Lex *et al*. 2014) with the ComplexUpset package (v1.3.3) running in R v4.4.2.

### Integration of panproteome and differential expression results

The homology table resulting from panproteome construction was integrated with differential expression (DE) analysis results by means of a custom script running in Python v3.10.9 and leveraging NumPY v1.24.1 (Harris *et al*. 2020), and Pandas v1.5.3 (McKinney 2010). The resulting homology/DE status table was further processed and visualized with a custom script running in R v4.2.2. A heatmap of non-ambiguously responding core groups was generated with the Pheatmap v1.0.12 package. After specific categories of homology groups (HGs) were selected, a Gene Ontology (GO) Biological Process (BP) enrichment analysis was performed for the *A. thaliana* gene identifiers present in said groups with TopGO v2.50.0 (Alexa *et al*. 2006), relying on the org.At.tair.db v3.16.0 Bioconductor annotation data package, running the “Classic” algorithm, and performing Fisher tests. Enrichment results for each set of groups were filtered by keeping only terms which were associated to at least 5 genes. A KEGG pathway enrichment analysis was subsequently performed on the *A. thaliana* genes present in the same categories of HGs with the enrichKEGG function of the ClusterProfiler v4.6.2 package (Yu *et al*. 2012; Wu *et al*. 2021). For both enrichment analyses, the set of background genes (i.e. the analysis “universe”) was composed by all *A. thaliana* genes surviving the DE analysis (i.e. genes for which an adjusted *P*-value could be calculated by DESeq2).

### Processing and visualization targeted analysis results

Expression profiles, TPM-normalized counts and homology relationships were processed and visualized with custom R scripts making use of packages dplyr (v1.1.0), ggplot2 (v3.4.1), janitor (v2.2.0), pheatmap (v1.0.12), scales(v1.2.1), stringr (v1.5.0), tidyr (v1.3.0). All scripts are available at https://doi.org/10.4121/5b88cdf2-eb5f-4033-8ece-1f3f488a1f83.

## Results

### Plant growth under a reliable high-light environment

In this study, we aimed to identify genes and pathways responsible for the higher photosynthetic LUE of *H. incana* under high-irradiance conditions. To create strongly contrasting growth conditions, we set our LL irradiance to $200\;\mu \mathrm{mol}\;{\text{m}}^{-2}\;{\text{s}}^{-1}$ and our HL irradiance to $1,800\;\mu \mathrm{mol}\;{\text{m}}^{-2}\;{\text{s}}^{-1}$ for 12h per day. We calculated ${\text{U}}_{2}$ irradiance uniformity values (Sun *et al*. 2014; Hu *et al*. 2015) over all growing positions designated for both treatments, and we selected positions on each growing table that resulted in the best irradiance uniformity. For the LL table, this resulted in an average irradiance of $227.5\;\mu \mathrm{mol}\;{\text{m}}^{-2}\;{\text{s}}^{-1}$ associated with an ${\text{U}}_{2}$ of 0.93, while for the whole HL table we measured an average irradiance of $1843.6\;\mu \mathrm{mol}\;{\text{m}}^{-2}\;{\text{s}}^{-1}$ , also associated with an ${\text{U}}_{2}$ of 0.93.

To compare the light treatments to conditions that plants would experience in natural environments, we calculated a daily light integral (DLI) (Faust and Logan 2018), a measure of the total irradiance delivered over the course of a day per unit of area, for each treatment. This resulted in DLIs of $9.82\;\mathrm{mol}\;{\text{m}}^{-2}\;{\text{d}}^{-1}$ and $79.64\;\mathrm{mol}\;{\text{m}}^{-2}\;{\text{d}}^{-1}$ for the LL and HL treatments, respectively.

**Fig. 1.**
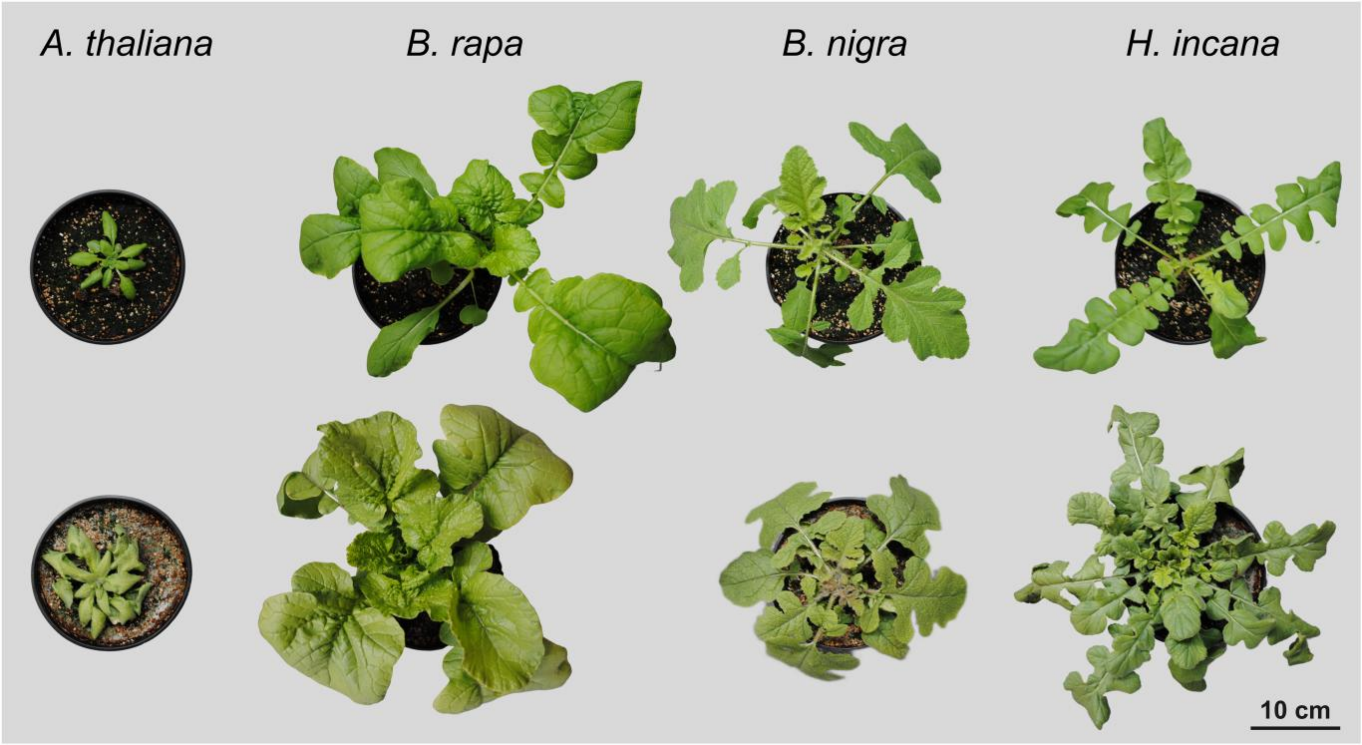
Pictures of representative plants at the end of the experiment for each of the 4 species grown under the 2 irradiance conditions. Left to right: *A. thaliana*, *B. rapa*, *B. nigra*, *H. incana*. Top row: LL irradiance. Bottom row: HL irradiance.

Besides *H. incana*, this study featured 3 other Brassicaceae species: *A. thaliana*, *B. rapa*, and *B. nigra*. These are the same we used for previous work in which they showed to have lower photosynthetic LUE than *H. incana* (Garassino *et al*. 2022). Plants of the 4 species established and grew without showing stress symptoms (Fig. 1, Supplementary Fig. S1), in line with what previously observed and measured on plants of the same species grown under the same conditions (Retta *et al*. 2024). However, 20% of the *B. nigra* plants from the LL treatment appeared to grow more slowly and had paler leaf color than the other *B. nigra* plants (Supplementary Fig. S1).

### Per-species differential expression analysis

To study the gene expression under contrasting light conditions in the 4 species, we sequenced 40 mRNA libraries (4species×  5replicates×2conditions) generated from RNA extracted from whole plant canopies, with an average of 22.2±2.4 million reads per library. The MultiQC inspection of all sequencing reads did not show any quality issues in our dataset. Percentages of reads mapped to reference genomes were high, ranging between 93.4±1.5% and 96.3±0.7% (Supplementary Table S1).

We performed DE analysis on data from each species individually with DESeq2 and selected all differentially expressed genes (Table 1, Supplementary Tables S2–S5). Per-species principal component analysis performed on regularized logarithm-transformed count data showed that the general patterns of gene expression are consistent across biological replicates belonging to the same species and originating from the same treatment (Supplementary Fig. S2). The percentages of genes significantly differentially expressed were similar for *A. thaliana*, *B. rapa*, and *H. incana*, while they were lower for *B. nigra* due to the high number of genes in the annotation. Since we are interested in differences and similarities between *A. thaliana* and the other species, and in particular *H. incana*, we compared the gene expression across the species.

**Table 1.** Numbers of differentially expressed and nondeferentially expressed genes for the various species.

	Number of genes		
	Higher expression	Lower expression	Unchanged expression
*A. thaliana*	3,346	3,027	21,226
	(12.1%)	(11.0%)	(76.9%)
*B. rapa*	7,292	7,138	32,050
	(15.7%)	(15.4%)	(68.9%)
*B. nigra*	4,723	4,052	50,934
	(7.9%)	(6.8%)	(85.3%)
*H. incana*	4,284	4,334	23,900
	(13.2%)	(13.3%)	(73.5%)

Percentages of the total number of genes are placed between brackets. Significant differences for P<0.05.

### Cross-species comparison using a panproteome

**Fig. 2.**
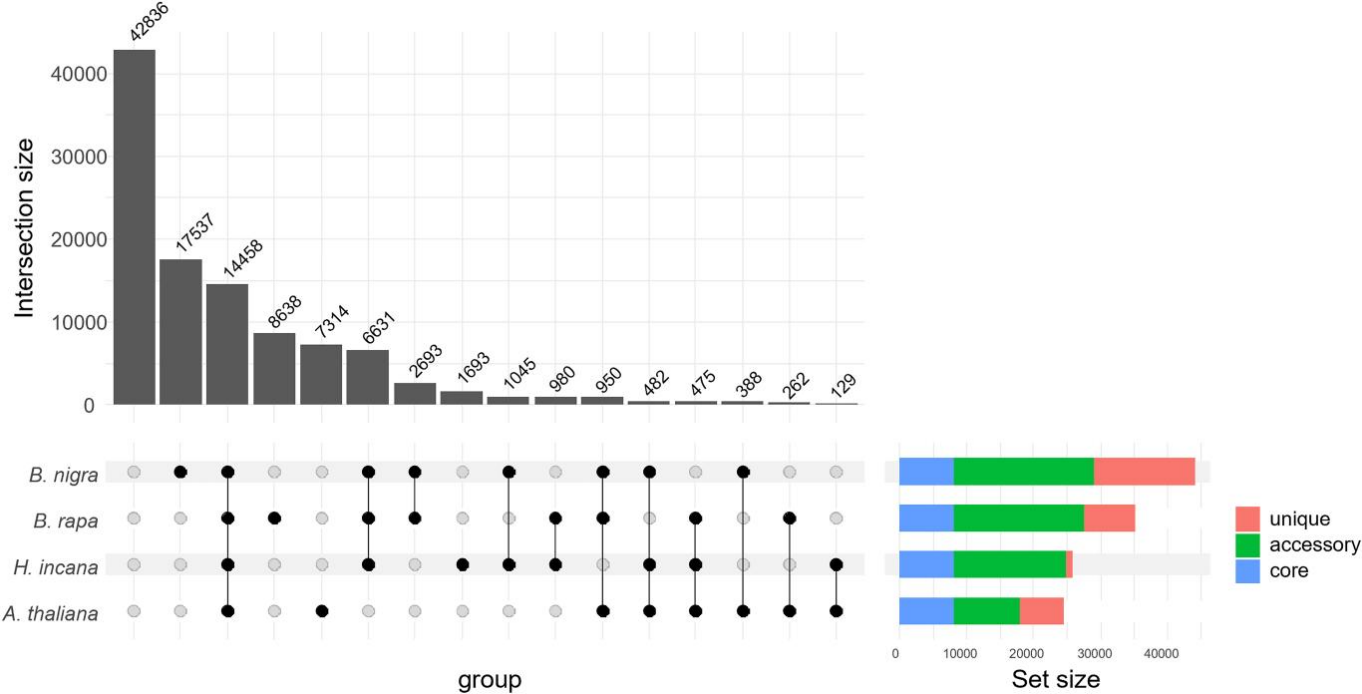
An UpSet plot of the panproteome HGs, based on the 4 species for which RNA-Seq was done. Vertical bars represent the number of HGs, classified by the presence/absence of genes from the various species as illustrated at the bottom of the figure. The first bar represents all the HGs of the panproteome constructed with proteomes from 8 Brassicaceae species that do not contain any genes from the 4 species we sequenced. The horizontal colored bars show how many of the HGs containing at least 1 gene from a species fall within 1 of 3 categories: core HGs (CHGs), i.e. those containing at least 1 gene from all 4 species; accessory HGs, i.e. those containing genes from more than 1, but not all, species; and unique HGs, i.e. those containing only genes from a single species.

To enable the comparison of gene expression across species, we built a panproteome to group homologous genes (orthologs and paralogs) (Sheikhizadeh Anari *et al*. 2018). A panproteome of 8 Brassicaceae species (*A. arabicum*, *A. thaliana*, *B. nigra*, *B. rapa*, *H. incana*, *R. raphanistrum*, *R. sativus*, *S. irio*) yielded 106,511 HGs (Supplementary Fig. S3 and Table S6). We then selected HGs containing at least 1 gene from 1 of the 4 species for which RNA-Seq was performed, leaving 63,675 HGs for downstream analysis (Fig. 2).

We also distinguished “differentially expressed” (DE) HGs, which contain at least 1 gene that was differentially expressed between both light conditions, and “non-DE” HGs (Table 2), which do not. Among the 19,012 DE HGs, the 10,770 which have an ortholog from each of the 4 species (i.e. the core proteome) form the main target of our research. Of particular interest are the differences and similarities between *A. thaliana* and the other species, all of which are members of tribe Brassiceae, and in particular *H. incana*. Similarly, we identified 3,688 HGs which contain genes that were not differentially expressed in any of the species and can, therefore, not explain the phenotypic differences (Supplementary Table S7). Based on the *A. thaliana* genes contained in these groups, we found 267 Gene Ontology (GO) Biological Process (BP) terms enriched (Supplementary Table S8). As expected, no terms related to photosynthesis or high-light adaptation were identified in this set of groups.

**Table 2.** Numbers of HGs in the constructed panproteome, classified based on their “DE” status.

	Homology groups		
	DE	Non-DE	Total
Core	10,770	3,688	14,458
Accessory	6,132	7,903	14,035
Unique	2,838	32,344	35,182
Total	19,012	44,663	63,675

An HG is classified as “DE” if it contains at least 1 gene differentially expressed between both light conditions. If this condition is not met, the HG is classified as “non-DE.”

To compare transcriptional activity across species within a single light condition, we compared TPM-normalized transcript counts. To assess bias due to differences in sequencing libraries, which are not corrected for during TPM normalization (Zhao *et al*. 2020), we tested whether average transcript abundances were similar across species. We selected the non-DE HGs containing a single expressed ortholog for each of the 4 species. We averaged TPM counts (regardless of treatment) for each species and calculated per-HG ${\text{log}}_{2}$-ratios between average counts for the single orthologs of the various species. The distribution of these ratios showed that on average the *A. thaliana* transcript abundances are higher than those of *B. rapa* and *H. incana* (% of area under the curve (AUC) for ${\text{log}}_{2}$-ratios >1:59.9and62.8%, respectively), which are in turn higher than those of *B. nigra* (% of AUC for ${\text{log}}_{2}$-ratios >1:67.4% for *B. rapa* and 67.2% for *H. incana*) (Supplementary Fig. S4 and Table S9). Given the fact that expression in *H. incana* is generally lower than in *A. thaliana* and similar to that in *B. rapa*, we conclude that detection of a significantly higher expression in *H. incana* is the effect of biological processes rather than an artifact.

### Comparative analysis of core DE homology groups highlights photosynthetic pathways

Of the 10,770 core HGs (CHGs) containing at least 1 gene differentially expressed under HL, 10,352 showed non-ambiguous DE within each species and were selected for downstream analysis. We defined non-ambiguous DE as the situation in which the expression of all genes is exclusively increased or decreased.

**Fig. 3.**
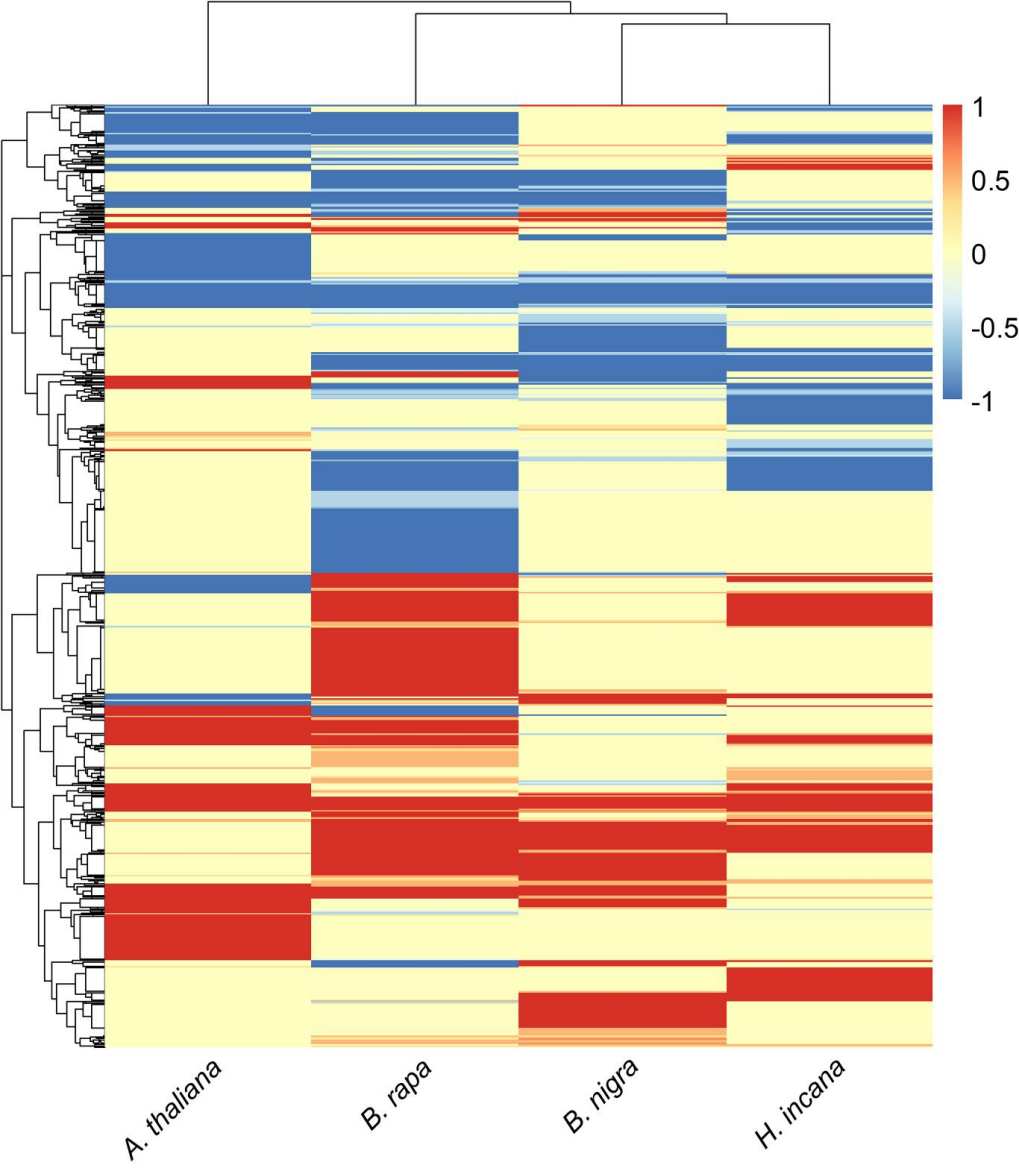
Heatmap of 10,352 groups showing only non-ambiguous responses per species. The color scale represents the ratio between the number of higher (positive numbers) or significantly lower expressed (negative numbers) genes and the total number of gene models present in each group per each species. Both rows and columns were clustered with hierarchical clustering based on Euclidean distances.

Clustering the CHGs with non-ambiguous responses allowed us to identify expression profiles for the 4 species (Fig. 3, Supplementary Table S10). Some CHGs show consistent higher or lower expression in all species (245 and 382, respectively). More often, an higher or lower expression is shared by some species (2,165 and 1,890 CHGs) or is unique to a species (2,163 and 1,946). Lastly, there are CHGs showing contrasting expression (higher expression in some species, lower expression in others) across species (1,561).

In order to get an overview of the role of the genes belonging to clusters of CHGs, we performed GO (Ashburner *et al*. 2000; Gene Ontology Consortium 2021) and KEGG (Kanehisa and Goto 2000; Kanehisa *et al*. 2023) enrichment analyses separately for clusters of CHGs containing at least 1 gene model with significantly higher or lower expression in each species, in the 3 Brassiceae species (*B. rapa*, *B. nigra*, *H. incana*), and in *H. incana* alone. GO enrichment analysis for the sets of *A. thaliana* genes in the clusters of CHGs containing genes with higher expression under HL resulted in terms related to response to water deprivation and salt stress, heat, low cellular oxygen, and flavonoid biosynthesis (Supplementary Table S11). A similar selection and analysis for genes with lower expression under HL resulted in terms involved in high-light response, chlorophyl metabolism, and growth regulation.

KEGG enrichment analysis highlighted 17 overrepresented pathways (Supplementary Table S12). Twelve pathways were enriched in CHGs containing at least 1 gene with higher expression under HL in all species, Brassiceae species, or *H. incana* alone. The most notable of these 12 pathways was “Carbon metabolism” (ath01200). The remaining 5 pathways were enriched in CHGs containing at least 1 gene with lower expression under HL in all species, Brassiceae species, or *H. incana* alone. Notably, these 5 pathways comprised the 2 currently annotated in KEGG for photosynthesis: “Photosynthesis” (ath00195), and “Photosynthesis—antenna proteins” (ath00196). Since all 3 photosynthesis-related KEGG pathways were highlighted by our enrichment analysis, we decided to further explore the expression of the genes associated with these pathways in search of clues on the higher photosynthetic LUE of *H. incana*.

### Targeted analysis of light-harvesting complex genes

**Fig. 4.**
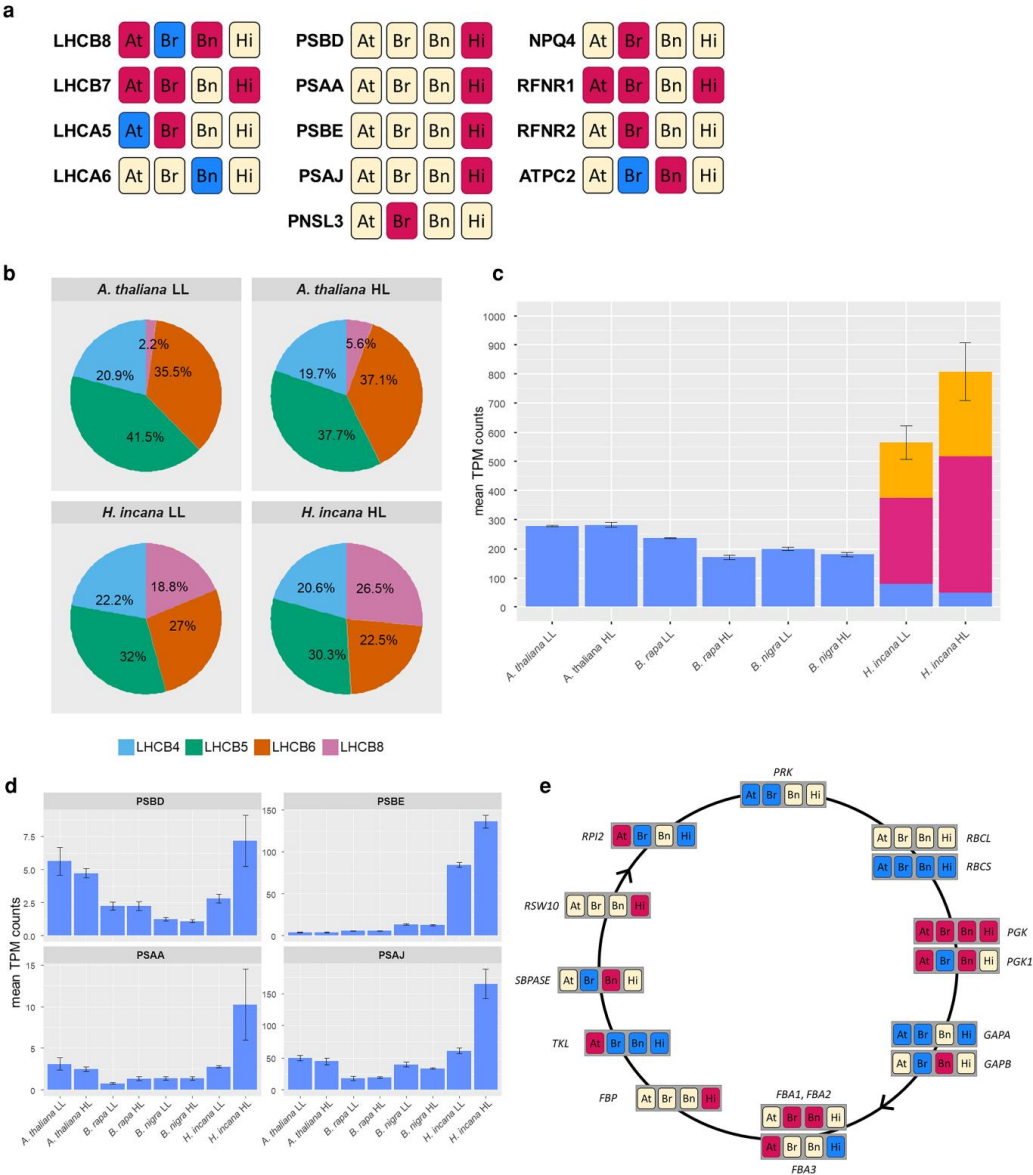
Results of the targeted analysis of photosynthetic pathways. a) Schematic view of the restricted set of genes belonging to KEGG pathways ath00196 (“Photosynthesis—antenna proteins,” left column) and ath00195 (“Photosynthesis,” center and right columns) showing higher expression under HL in at least one of the species. Magenta box indicate significantly higher expression under HL, while blue boxes indicate significantly lower expression under HL. At: *A. thaliana*; Br: *B. rapa*; Bn: *B. nigra*; Hi: *H. incana*. b) Pie charts representing the relative abundance of transcripts originating from genes encoding components of the PSII minor antenna. c) Mean normalized abundance of *LHCA6* transcripts in plants of the 4 species grown under the 2 irradiance treatments. The different colors represent different paralogs. Error bars represent the standard errors of the mean. The full comparison of transcript abundances for *LHCA6* can be found in Supplementary Fig. S7. d) Mean normalized abundances of *PSBE*, *PSBD*, *PSAA*, and *PSAJ* transcripts in plants of the 4 species grown under the 2 irradiance treatments. Error bars represent the standard errors of the mean. Full across-species comparisons can be found in Supplementary Figs. S9, S10, S11, and S12. e) Schematic representation of the Calvin–Benson cycle, or the “dark reactions” of photosynthesis, and DE status of genes involved in the 4 species. Yellow: no significant DE under HL; red: significantly higher expression under HL; blue: significantly lower expression under HL. At: *A. thaliana*; Br: *B. rapa*; Bn: *B. nigra*; Hi: *H. incana*.

We first analyzed the expression patterns of the *A. thaliana* genes annotated with the KEGG pathway “Photosynthesis—antenna proteins” (ath00196) and their orthologs in *B. rapa*, *B. nigra*, and *H. incana*. This allowed us to investigate transcriptional differences associated with light-harvesting complexes (LHCs), which are amongst the first complexes involved in the photosynthetic process. The KEGG pathway is made up of 22 *A. thaliana* genes assigned to 14 HGs. These groups contain 34 genes for *B. rapa*, 33 genes for *B. nigra*, and 35 genes for *H. incana*. Inspection on these genes revealed ${\text{log}}_{2}$ fold change (${\text{log}}_{2}$FC) values ranging between −3.15 and 1.01, with almost all genes showing significant lower expression under HL, except for *LHCB8*, *LHCB7*, and *LHCA5* (Supplementary Fig. S5). No differences across species were observed except for 2 genes coding for photosystem II (PSII) antenna proteins, *LHCB8* and *LHCB7*, and 2 coding for photosystem I (PSI) antenna proteins, *LHCA6* and *LHCA5* (Fig. 4a). Considering the particular features explained below, we selected the *LHCB8* and *LHCA6* genes for further investigation.

*LHCB8* was first investigated as member of a subset of rarely expressed LHC protein encoding genes (Klimmek *et al*. 2006). The LHCB8 protein resembles the CP29.1 and CP29.2 proteins, encoded in *A. thaliana* by the *LHCB4.1* and *LHCB4.2* genes, and is, therefore, also known as CP29.3 (*LHCB4.3*). *AtLHCB8* shows a different expression pattern than *AtLHCB4.1* and *AtLHCB4.2*, suggesting a different role for the protein in the LHC. It seems to be present only in species of the eurosids, a subclade of the rosids (Klimmek *et al*. 2006). The LHCB8 protein is present as a monomer within the PSII supercomplex, forming the so-called “minor antenna” of PSII with a number of other LHCB proteins (LHCB4, LHCB5, LHCB6) (de Bianchi *et al*. 2011). The expression of *A. thaliana LHCB8* is induced by high-irradiance conditions (Flannery *et al*. 2021a), and a substantial increase in abundance of the LHCB8 protein was reported in pea plants grown under high irradiance (Grinzato *et al*. 2020). *LHCB8* is a single-copy gene in the 4 species used for this study and under HL had moderately higher expression in *A. thaliana* and *B. nigra*, while it had moderately lower expression in *B. rapa* and showed no significant changes in *H. incana*. Based on TPM-normalized read counts, *LHCB8* transcripts represent roughly 26.5% of the transcript pool for the minor antenna in *H. incana* plants grown under HL, while they represent only 5.6%, in *A. thaliana* (Fig. 4b). This high representation is also found in *B. rapa* and *B. nigra*, with *LHCB8* making up 22.5% and 31.1% of minor antenna transcripts (Supplementary Fig. S6).

*LHCA6* is a poorly-expressed gene coding for a protein associated with PSI as an antenna monomer. *LHCA6* is present as a single-copy gene in *A. thaliana*, *B. rapa*, and *B. nigra*, while it has 3 tandem copies in *H. incana* (Garassino *et al*. 2022). *LHCA6* did not show statistically significant changes in expression in *B. nigra*, *A. thaliana* and in any of the 3 copies of *H. incana*. However, it had lower expression in *B. rapa* under HL. To determine expression across species, we summed TPM-normalized counts for the 3 *LHCA6* copies in *H. incana* and calculated all pairwise ratios between counts in the 4 species under the 2 treatments. Inspection of ratios between counts in *H. incana* and other species revealed the *LHCA6* paralogs to have higher expression in *H. incana* under both the irradiance conditions after correction for the transcriptional baseline differences (Fig. 4c, Supplementary Fig. S7).

### Targeted analysis of light reactions genes

We then analyzed the expression patterns for the CHGs containing the 77 *A. thaliana* nuclear and chloroplast genes that are annotated with the KEGG pathway “Photosynthesis” (ath00195), which are involved in the light reactions of photosynthesis. ${\text{Log}}_{2}$FC values ranged between 1.98 and −1.78, with 142 of the total 368 genes significantly DE under HL. When considering only the significant expression changes, the trend across “Photosynthesis” pathway genes is lower expression under HL in all species: 29 genes out of 33 in *A. thaliana*, 48 out of 52 in *B. rapa*, 25 out of 26 in *B. nigra*, and 27 out of 31 in *H. incana* (Supplementary Fig. S8). A small number of CHGs contained at least 1 gene having higher expression under HL in one of the species (Fig. 4a). For further analysis, we focused on those showing higher expression only in *H. incana*, thus selecting genes *PSBD* and *PSBE*, part of the PSII complex, and *PSAA* and *PSAJ*, part of the PSI complex.

The D2 protein, encoded by *PSBD*, forms the core of PSII along with the D1 protein, encoded by *PSBA*. These 2 subunits together bind 3 macromolecules that are fundamental for photosynthetic light reactions: the P680 reaction center, which transfers energy to water molecules, the $Mn_{4}\mathrm{Ca}O_{5}$ cluster responsible for the splitting of water molecules and retrieval of electrons, and components of the primary electron transfer chain, such as plastoquinones ${\text{Q}}_{A}$ and ${\text{Q}}_{B}$ (Leegood 2013). The PSII reaction center is completed by the subunit encoded by the *PSBI* gene and cytochrome b559, composed of subunits encoded by the *PSBE* and *PSBF* genes and a heme cofactor (Johnson and Pakrasi 2022). The *PSBD* gene is highly expressed in *A. thaliana* plants grown under both treatments, and *H. incana* plants grown under HL, while the *PSBE* gene is highly expressed only in *H. incana* plants grown under HL (Fig. 4d, Supplementary Figs. S9 and S10).

The PSI core is composed of proteins encoded by the *PSAA* and *PSAB* genes. The PSI complex is composed of several additional subunits, including one stabilized by the protein encoded by gene *PSAJ* (Scheller *et al*. 2001). The expression of *PSAA* and *PSAJ* orthologs appears to be significantly higher in *H. incana* plants grown under HL, with plants growing under LL having similar transcript levels to those measured in the other species irrespective of the treatment (Fig. 4d, Supplementary Figs. S11 and S12).

### Targeted analysis of carbon metabolism genes

Continuing our analysis based on photosynthesis KEGG-related pathways, we studied the expression of the 273 *A. thaliana* nuclear and chloroplast genes associated to the KEGG pathway “Carbon metabolism” (ath01200) and their orthologs (Supplementary Fig. S13). Pathway ath01200 comprises genes involved in both catabolism and anabolism of carbon-based molecules, organized in a number of modules. Inspection of these modules revealed that the genes related to the Calvin–Benson cycle, and thus to assimilation of inorganic carbon into the end product of photosynthetic reactions, carbohydrates, were grouped into module “Reductive pentose phosphate cycle (Calvin cycle)” (ath_M00165). The expression of genes included in this module did not show an obvious profile (Supplementary Fig. S14). However, 2 of the 23 CHGs associated with this module contained genes that had higher expression uniquely in *H. incana* under HL. These are orthologs of the *A. thaliana* genes *FBP* and *RSW10* (Fig. 4e). *A. thaliana* mutants for the *RSW10* gene has been linked to ribose-5-phosphate metabolism and cellulose biosynthesis, but no direct involvement with photosynthetic activity has been described to date (Howles *et al*. 2006; Xiong *et al*. 2009). Gene *FBP*, instead, has been associated with photosynthetic activity, and *FBP* overexpression, combined with that of the chloroplast envelope triose phosphate/phosphate translocator (*TPT*), has been proven to increase soluble sugar and starch contents, as well as photosynthetic $CO_{2}$ assimilation (Cho *et al*. 2012).

## Discussion

In this study, we explored the transcriptomes of plants of 4 Brassicaceae species (*A. thaliana*, *B. rapa*, *B. nigra*, *H. incana*) grown under contrasting irradiances to unravel the genetic determinants of *H. incana*’s high photosynthetic LUE under high irradiance. Considering the complexity of our dataset and based on the results of our untargeted enrichment analysis, we decided to restrict our exploration by focusing on genes related to photosynthesis in the KEGG ontology.

### Increasing power of elimination through super-natural irradiance

To make sure we would observe any transcriptional differences associated with growth under high-irradiance conditions, we designed and built a high-output, high-uniformity lighting system. The per-treatment DLI of $9.82\;\mathrm{mol}\;{\text{m}}^{-2}\;{\text{d}}^{-1}$ that we measured for the LL treatment $\left(227.5\;\mu \mathrm{mol}\;{\text{m}}^{-2}\;{\text{s}}^{-1}\right)$ is consistent with what has been reported for winter months in warm-temperate climate areas, while the DLI of $79.64\;\mathrm{mol}\;{\text{m}}^{-2}\;{\text{d}}^{-1}$ we measured for the HL treatment ($1843.6\;\mu \mathrm{mol}\;{\text{m}}^{-2}\;{\text{s}}^{-1}$) is substantially higher than the values of $60-65\;\mathrm{mol}\;{\text{m}}^{-2}\;{\text{d}}^{-1}$ reported for summer months in the same climate areas (Korczynski *et al*. 2002; ENEA TER-SOLTERM 2006; Faust and Logan 2018; Australian Government BoM 2022), or than the values of $58-64\;\mathrm{mol}\;{\text{m}}^{-2}\;{\text{d}}^{-1}$ reported for the summer solstices at the Equator and Tropics (Ritchie 2010). The DLI and the associated irradiance of our HL treatment can, therefore, be defined as “super-natural,” but are not unreasonable for plant growth in controlled conditions (i.e. with no nutrients and/or water limitations) (Warrington and Norton 1991; Song *et al*. 2018).

Our study differs from previous studies on HL responses not only because of our use of the super-natural magnitude of our HL treatment, but also for the way the treatment was applied. While previous high-light studies involving *A. thaliana* have employed irradiances ranging from between 150 and 2,000 $\mu \mathrm{mol}\;{\text{m}}^{-2}\;{\text{s}}^{-1}$, all of these studies applied the HL treatment to LL-adapted plants and focused on the response, or acclimation, to the HL (Caldana *et al*. 2011; Page *et al*. 2012; Bode *et al*. 2016; van Rooijen *et al*. 2018; Huang *et al*. 2019; Alvarez-Fernandez *et al*. 2021; Tiwari *et al*. 2021; Bobrovskikh *et al*. 2022; Pech *et al*. 2022). We, on the other hand, focused on the steady-state transcriptional activity in the 4 species we examined grown from the seedling stage to maturity under either low or high light.

We have shown that between 68.9% and 85.3% of genes from the 4 species were not differentially expressed between the light treatments (Table 1). Furthermore, after performing homology grouping and integrating its results with gene DE analysis we identified a total of 44,663 HGs containing genes that did not respond to the treatment (Table 2), as well as 631 out of the total 10,352 CHGs containing genes that have the same response to the treatment in all species (Fig. 3). None of these genes can, therefore, cause the higher photosynthetic LUE under HL of *H. incana*, and were, therefore, not considered in our further analysis. We thus believe that the combination of magnitude and application of treatment in our study gives us a sizeable “power of elimination” when dealing with complex transcriptomic datasets.

### Dealing with the complexity of across-species transcriptomic comparisons

The major challenge faced in comparative transcriptomics studies is the identification of homology relationships between the transcriptomes of different species. In contrast to previous studies (Bräutigam *et al*. 2011; Gowik *et al*. 2011; Aubry *et al*. 2014; Rao *et al*. 2016; Kümpers *et al*. 2017; García de la Torre *et al*. 2021; Julca *et al*. 2021; Curci *et al*. 2022; Wu *et al*. 2022), we inferred between-species transcript homology relationships by using a panproteome built with PanTools (Sheikhizadeh *et al*. 2016) to infer gene homology relationships. We made use of optimized homology grouping, based on the organization of universal single-copy orthologs (BUSCO gene sets, Manni *et al*. 2021a, 2021b) that is unique to PanTools (Sheikhizadeh Anari *et al*. 2018; Jonkheer *et al*. 2022). This method determines the optimal strictness of protein-clustering settings, given the phylogenetic distance between the proteomes in the dataset.

Integrating HGs with per-species transcript abundance and DE data presented us with the challenge of comparing transcript abundances across species. Canonical normalization methods, such as the TPM normalization we used in our study, do not yield abundance measures that can be compared between species (Zhao *et al*. 2020). In the absence of a widely accepted approach to compare normalized transcript abundances across species, we decided to estimate the transcriptional “baseline” of the 4 species. We extracted expression data for all the nondifferentially expressed (non-DE) genes belonging to single-copy CHGs and calculating gene-by-gene ${\text{log}}_{2}$-ratios between transcript abundances. Inspection of the distributions of these ratios revealed that *A. thaliana* has on average a slightly higher transcriptional baseline than *B. rapa* and *H. incana*, which in turn have a slightly higher baseline than *B. nigra*. We decided to control for these differences when comparing transcript abundance across species by calculating pairwise ${\text{log}}_{2}$-ratios between TPM-normalized transcript counts and relating them to the ratios calculated for non-DE genes. As we have shown for the genes highlighted in the pathway analyses, the differences between TPM counts are much larger than what could be explained by differences in “baseline” transcription (Supplementary Figs. S7, S9–S12), and therefore have biological meaning.

### Across-species comparison of differential gene expression highlights differences in photosynthetic pathways

The analysis of DGE, we performed individually on all 4 species in this study revealed similar percentages of DE genes for *A. thaliana*, *B. rapa*, and *H. incana*. Indeed, the cumulative percentage of DE genes in response to HL ranged between 23% and 31% (Table 1). This is in line with previous studies reporting that roughly 20% of the *A. thaliana* transcriptome is responsive to light (Ruckle *et al*. 2012; Bode *et al*. 2016). For *B. nigra*, on the other hand, only about 15% of the genes were DE under HL. We do not believe that this difference has a biological explanation, but that it is the result of the very large number of gene models included in the *B. nigra* annotation. Many of these gene models are likely to be annotation artifacts rather than actual genes, as shown by the large number of *B. nigra* genes clustering separately from genes of the other species in the panproteome (Fig. 2, Supplementary Fig. S3).

After quantifying gene expression for our 4 species and inferring homology relationships between genes, we performed a number of untargeted analyses aimed at giving us a nonbiased overview of the biological processes and pathways most affected when comparing the transcriptomes from the LL and HL treatments. By making use of the panproteome, we were able to perform these analyses on specific groups of genes, namely the CHGs containing DE genes in all 4 species combined, the Brassiceae species (*B. rapa*, *B. nigra*) and *H. incana* as a group, and *H. incana* on its own. Considering the higher photosynthesis rates we previously reported for the Brassiceae species (Garassino *et al*. 2022), one might expect results linked to photosynthetic LUE to come from the HGs showing DE for the Brassiceae species, or from the HGs with genes showing DE in *H. incana* alone. Nevertheless, the most promising results came from enrichment analyses on the HGs containing genes deferentially expressed across all 4 species. Indeed, out of a total of 9 KEGG pathways enriched in this kind of HGs, 3 pathways mentioned photosynthesis in their name.

One striking finding of our targeted analysis of the 3 photosynthesis-related pathways was that most of the associated genes appeared to either have lower or unchanged expression under the HL treatment. This was expected for the “Photosynthesis—antenna proteins” pathway (ath00196), including all photosystem antenna genes, based on experimental evidence that plants growing under HL will reduce the size of their antennas (Ballottari *et al*. 2007). However, this trend of lower or unchanged gene expression was unexpected for genes related to photosynthetic light reactions (included in the “Photosynthesis” pathway, ath00195) and carbon metabolism (included in the homonymous pathway, ath01200). Recent studies of changes in the *A. thaliana* proteome in response to irradiance increase or switch from controlled to field conditions have highlighted increases in abundance for most proteins involved in light reactions (Flannery *et al*. 2021a, 2021b). Furthermore, ample experimental evidence has been collected in the past showing that plants acclimating to HL develop a higher carbon fixation metabolism via increased protein levels (Schöttler and Tóth 2014). A few considerations arise from the discrepancy between this evidence and the results of our transcriptome analysis. The first is that, as already discussed above, previous studies focused on acclimation responses to higher light, while ours was conducted on plants that grew under constant high or LL. Therefore, the transcriptome snapshot obtained in our study is the representation of a possibly different gene and protein regulation, related more to the maintenance of photosynthesis under high irradiance rather than to the response to an important change in irradiance conditions as studied in previous research. Furthermore, it is important to point out that while transcriptome analysis highlights genes that are potentially involved in high photosynthetic LUE, it cannot inform us on downstream proteome dynamics. Thus, we currently cannot say whether higher gene expression is a consequence of higher protein turnover due to e.g. photodamage, or if it enables for higher protein abundance, thus potentially enabling for higher biochemical capacity in the photosynthetic reactions. The opposite is naturally true for lower gene expression, and therefore this analysis does not allow us to conclude whether that is the result of higher protein stability or lower protein abundance requirements.

Finally, our in-depth analysis of gene expression for 3 KEGG pathways revealed that DGE is only one of the ways *H. incana* achieves higher transcript abundances, potentially enabling its higher photosynthetic LUE. While we identified 4 genes encoding photosystem subunits (*PSBD*, *PSBE*, *PSAA*, *PSAJ*) whose transcript levels were significantly higher in *H. incana* plants grown under HL, we identified other genes such as *LHCB8* and *LHCA6* having a striking transcript abundance in *H. incana* plants grown under both irradiances. This appears to be achieved in 2 additional ways: while the *LHCB8* gene is present in a single copy in *H. incana* and all other species, and the abundance of its transcript in *H. incana* can be explained with a constitutive overexpression of the gene, the *LHCA6* gene is present in 3 copies in *H. incana* as opposed as the single copy of the other 3 species. Each *LHCA6* copy is expressed in *H. incana* at levels that appear to be slightly higher than those of other species, but the cumulative expression of the 3 copies results in a substantially higher transcript abundance for the gene. These strategies to achieve higher gene expression form an interesting lead to further investigate the precise relationship between expression levels, protein abundance and turnover, and ultimately the photosynthetic LUE of *H. incana*.

### Possibilities to explore other processes related to photosynthesis

While we decided to limit our research to KEGG photosynthesis pathways, we acknowledge that photosynthesis is a highly complex process involving other key pathways. We hypothesize that genes involved in transpiration, heat dissipation, stress response, and nutrient uptake and cycling will play a role in supporting higher photosynthetic efficiency. While previous studies identified transcriptional responses to high irradiance connected to heat-shock response (van Rooijen *et al*. 2018; Bobrovskikh *et al*. 2022), ribosome biogenesis and transcriptional activity (Bobrovskikh *et al*. 2022), lipid remodeling (van Rooijen *et al*. 2018), flavonoid biosynthesis (Page *et al*. 2012; Pech *et al*. 2022), a comprehensive picture of these responses is still far from being available (Burgess *et al*. 2023). Based on what emerged from our targeted analysis on photosynthetic pathways, approaching our dataset in a different way than via enrichment analysis will likely reveal how the these processes are playing a role in high-light photosynthesis. Our resource will, therefore, provide means to further explore the genetic basis of high photosynthetic efficiency under HL.

### Prospects for future research

In this study, we have highlighted 3 different strategies that *H. incana* can employ to achieve higher transcript abundances for genes that potentially play a key role in its photosynthetic efficiency. Given that our analysis pipeline allows the retrieval of TPM-normalized counts for all expressed genes in each of the employed species, and that we established a method to estimate baseline differences in transcript abundances for the various species, an additional study of transcript abundances irrespective of DE might provide further clues on the mechanisms allowing *H. incana* to achieve higher photosynthetic LUE. Despite showing that most genes involved in photosynthetic reactions have lower expression as a response to HL in all analyzed species, we have identified a number of genes that are either highly expressed in response to HL or have a constitutive higher expression in *H. incana*. Of these genes, *LHCB8* and *LHCA6* appear as very promising targets for further analysis, as the function of the first is still unclear and the higher expression in *H. incana* of the second cannot be explained with current literature.

Furthermore, the dataset we have generated can be analyzed from other perspectives. As an example, broader explorations focusing on maintenance of the photosynthetic machinery under high irradiance could be performed across the 4 species. These could result in a more generalized understanding of photosynthetic performance under high irradiance, which we see as a natural complement to the understanding of the performance of high-LUE species *H. incana* which we targeted in this work.

It is important to stress once more how this experiment aimed at obtaining a snapshot of the operation of high photosynthesis rates, rather than at their establishment during leaf development. While our experiment uncovered some genes that might be playing a role in supporting high photosynthetic activity under high irradiance, future transcriptomics investigations on time series collected throughout leaf development will be crucial to understand which genes and processes enable the establishment of high photosynthetic LUE.

### Conclusion

This study provides an analysis of the transcriptomes of *A. thaliana*, *B. rapa*, *B. nigra*, and *H. incana* plants grown under constant low and high irradiance, rather than the acclimation response to high irradiance. By combining gene expression quantification and DE analysis with a panproteome-based homology grouping, we quickly and efficiently identified expression patterns shared by the various species, or unique to one of them. Following an untargeted approach, we observed an enrichment for genes involved in photosynthetic pathways. A closer look at the expression of all genes belonging to these pathways allowed us to reveal that in comparison to other Brassicaceae species, *H. incana* growing under a HL treatment achieves higher expression of genes related to photosynthesis via 3 different modes: “canonical” DE between low and HL, constitutive higher expression of single-copy genes, or cumulative higher expression obtained by simultaneous expression of multiple gene copies. Besides identifying genes such as *LHCB8* and *LHCA6*, whose higher expression in *H. incana* growing under HL prompts for a detailed investigation of their role in photosynthetic LUE under high irradiance, we believe that analyzing the genes undergoing DE specifically in *H. incana* will further clarify the role of nonstrictly photosynthetic genes in supporting the species’ striking photosynthetic performance. Therefore, we expect the resource we established with this study to provide further, extensive knowledge on the genetic strategy employed by *H. incana* to support its high photosynthetic LUE.

## Supplementary Material

jkae175_Supplementary_Data

## Data Availability

Raw sequencing data have been deposited to NCBI and can be found under BioProject PRJNA1001172. All scripts used for data analysis are available on the 4TU.ResearchData platform with the DOI https://doi.org/10.4121/5b88cdf2-eb5f-4033-8ece-1f3f488a1f83. A data package with supplementary information containing Supplementary Tables S1–S19, count tables, and filtered annotations is available on the 4TU.ResearchData platform at DOI https://doi.org/10.4121/d3455b3c-54d8-4ef8-8501-a70936a51dad. Supplemental material available at G3 online.
